# Supplementary material for: Visual field inhomogeneities and the architectonics of early visual cortex shape visual working memory
Source: Cereb Cortex. 2026 May 22;36(5):bhag058. doi: 10.1093/cercor/bhag058 (PMC13196602; doi:10.1093/cercor/bhag058)
Supplement: Supplementary_materials_final_bhag058 [file supplementary_materials_final_bhag058.docx]

### **Supplementary materials**

### **Visual Field Inhomogeneities and the Architectonics of Early Visual Cortex Shape Visual Working Memory**

Julia Papiernik-Kłodzińska^1,2,3*^, Simon Hviid Del Pin^4^, Kristian Sandberg^5,6^, Michał Wierzchoń^1,3^, Marisa Carrasco^7^, & Renate Rutiku^1,3*^

^1^ Consciousness lab, Institute of Psychology, Jagiellonian University, Kraków, Poland

^2^ Doctoral School in the Social Sciences, Jagiellonian University, Kraków, Poland

^3^ Centre for Brain Research, Jagiellonian University, Kraków, Poland

^4^ Department of Computer Science, Norwegian University of Science and Technology, Gjøvik, Norway

^5^ Center of Functionally Integrative Neuroscience, Department of Clinical Medicine, Aarhus University and Aarhus University Hospital, Universitetsbyen, Aarhus, Denmark;

^6^ Neurobiology Research Unit, Department of Neurology, Copenhagen University Hospital Rigshospitalet, Copenhagen, Denmark;

^7^ Department of Psychology and Center for Neural Science, New York University, New York, NY, USA

**Corresponding authors:**

Julia Papiernik-Kłodzińska, Consciousness Lab, Institute of Psychology, Jagiellonian University, 6 Ingardena Street, 30-060 Krakow, Poland. Email: julia.papiernik-klodzinska@uj.edu.pl

Renate Rutiku, Consciousness Lab, Institute of Psychology, Jagiellonian University, 6 Ingardena Street, 30-060 Krakow, Poland. Email: [renate.rutiku@uj.edu](mailto:renate.rutiku@uj.edu)

1. Experimental stimuli

The stimuli for the task were chosen from the Snodgrass and Vanderwart *Like* object repository (Rossion and Pourtois 2004); for details, see the methods section). A randomly chosen subset of experimental stimuli is presented in Figure S1.


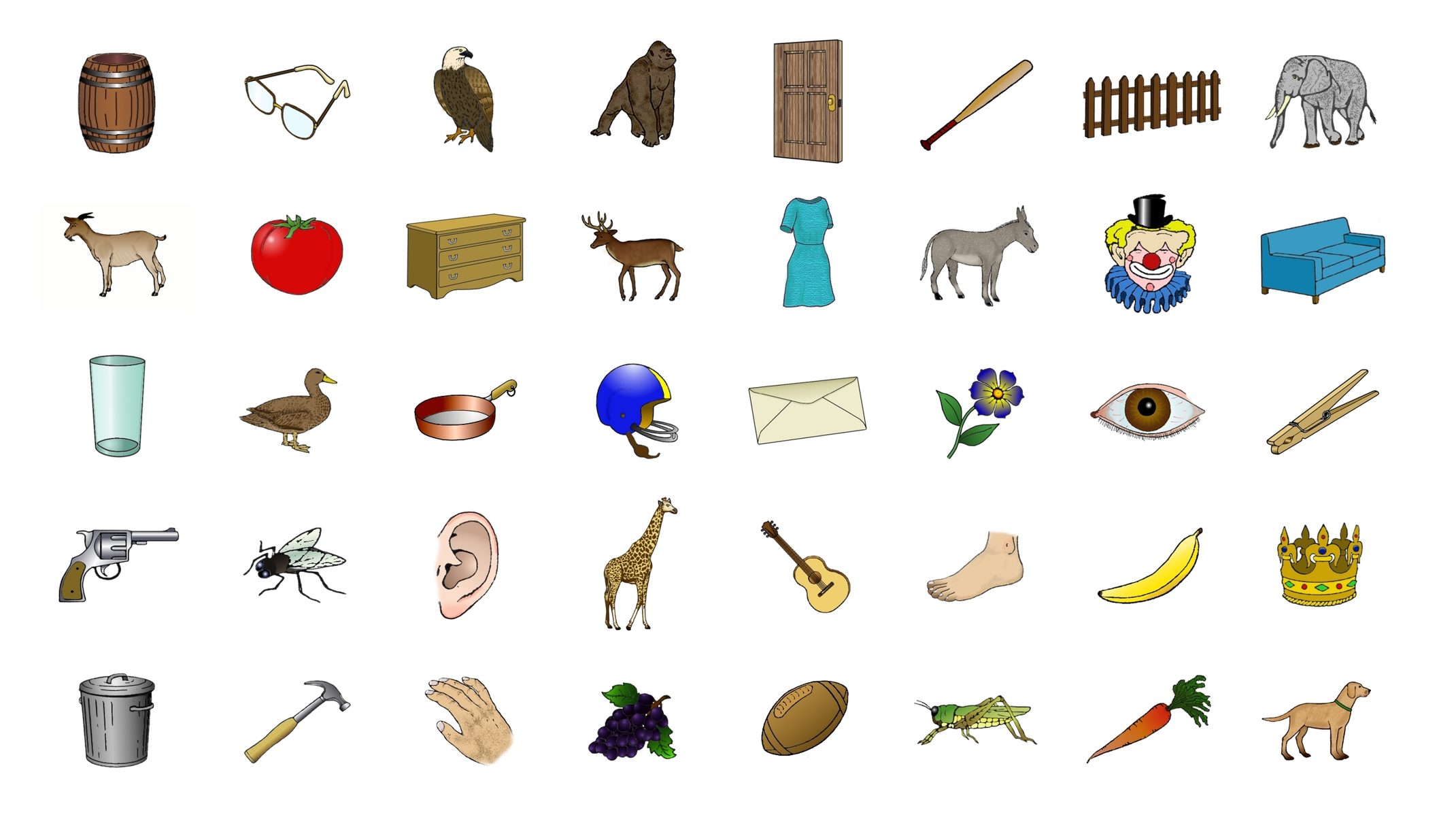


*Figure S1*. A randomly chosen subset of experimental stimuli,

1. Stimuli type effect on performance

A paired t-test was performed to check whether objects from one category (manmade or natural) were perceived with higher accuracy. The results show that responses to natural stimuli were more often correct than responses to manmade stimuli (t = 3.8, p <0.001, Cohen’s d = 0.54, df = 198), with average accuracy rate reaching 0.74 for natural, and 0.71 for manmade objects (SD = 0.05 and 0.04, respectively). The tendency for higher accuracy for natural stimuli was consistent across locations (see Figure S2B), yet any specific participants did not carry the results; as presented in Figure S2C, participants who tended to have higher accuracy in natural stimuli tended to have a high accuracy for the manmade stimuli as well. This was confirmed by Pearson's correlation (r(260) = 0.57, p < 0.001). The average accuracy for each stimulus used in the procedure is presented in Figure S2


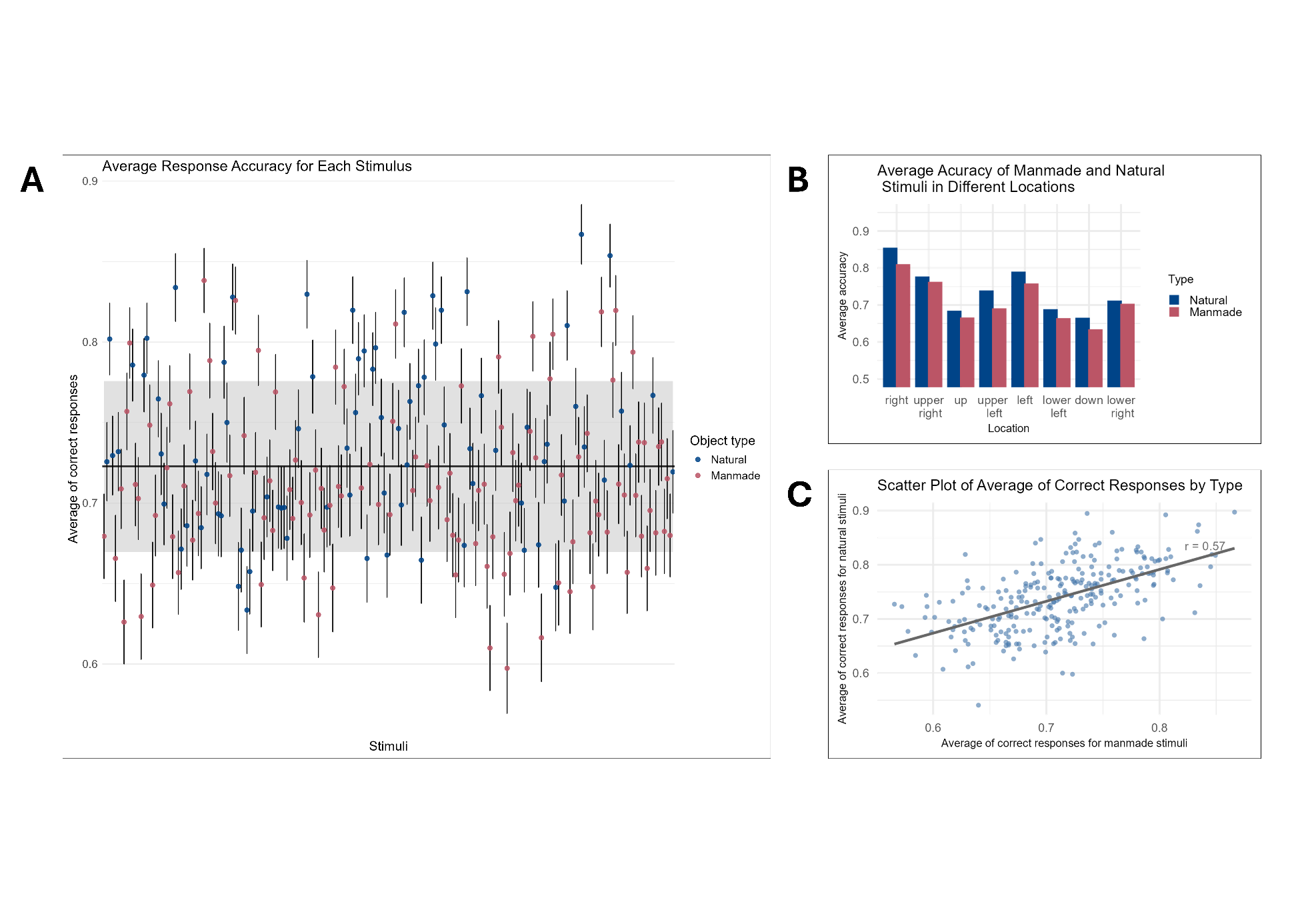


*Figure S2*. Average response accuracy for each stimulus used for the procedure. The naturally occurring objects are marked in blue, and the manmade in red. The thin black lines represent the standard error for the average response accuracy for each stimulus across participants. The thick black line represents the average accuracy across all objects, and the gray shading – the confidence interval (M = 0.71, SD = 0.05)

1. Eye dominance and height

The effects of eye dominance and height on the asymmetry indices were tested in order to assess whether they could confound the data. The reasoning behind testing the link between the height on the indices was that the vertical meridian anisotropy is known to vary between children and adults, (Carrasco et al. 2022) appearing and developing throughout adolescence (Carrasco et al. 2023; Carrasco et al., 2025), which is believed to be linked to the development of the preference for perceiving the lower half of the visual field, as this part of the visual field tends to hold relevant information on a day-to-day basis (Carrasco et al., 2023). For completeness, we performed Pearson’s correlation tests for the effect of height on all asymmetries, yet the results were null, consistent with previous research (Carrasco et al., 2023; see: Table S1).

The effect of eye-dominance on the asymmetry indices was tested post-hoc after the data analysis showed that our participants had higher accuracy for recognizing objects appearing in the right visual field. However, the Kruskal-Wallis tests yielded null results (see Table S2).

|  | **r(260)** | **t** | **p-value (uncorrected)** | **p-value (corrected)** |
| --- | --- | --- | --- | --- |
| Horizontal-Vertical Anisotropy | 0.13 | 2.11 | 0.04 | 0.14 |
| Vertical Meridian Asymmetry | -0.01 | -0.18 | 0.85 | 0.85 |
| Upper-lower VFI | 0.04 | 0.62 | 0.53 | 0.71 |
| Left-right VFI | 0.05 | 0.74 | 0.46 | 0.71 |

*Table S1.* Pearson’s correlation between individual asymmetry indices and height of the participants. The p-values were corrected using the False Discovery Rate method.

|  | **χ2 (1)** | **p-value (uncorrected)** | **p-value (corrected)** |
| --- | --- | --- | --- |
| Horizontal-Vertical Anisotropy | 0.36 | 0.55 | 0.79 |
| Vertical Meridian Asymmetry | 1.04 | 0.31 | 0.79 |
| Upper-lower VFI | 0.14 | 0.7 | 0.79 |
| Left-right VFI | 0.07 | 0.79 | 0.79 |

*Table S2.* Kruskall-Wallis test results of the effect of eye dominance on individual indices.

1. On- and non-meridian asymmetries

The visual field analysis of the on-meridian upper versus lower visual field (*up* vs *down*) demonstrated a small effect size (t = 7.5, df = 261, Cohen’s d = 0.6, p<0.001), while the non-meridan upper versus lower visual field analysis (mean(*upper right, upper left*) vs mean(*lower right, lower left*)) showed a smaller t value, but a medium effect size (t = 6.88, df = 261, Cohen’s d = 0.56, p<0.001). Both isocentric (*left* vs *right*) and non isocentric (mean(*lower left, upper left*) vs mean(*lower right, lower right*)) left versus right visual field analyses showed a small effect size. (for on-axis: t = -747, df = 261, Cohen’s d = 0.-0.53, p<0.001; for off-axis: t = 5.52, df = 261, Cohen’s d = 0.42, p<0.001). The summary of comparisons of different visual field effects can be found in Table S3.

|  | **t(261)** | **p uncorrected** | **p corrected** | **Cohen’s d** |
| --- | --- | --- | --- | --- |
| Upper-lower VF vs vertical axis | -0.79 | 0.42 | 0.43 | -0.04 |
| Left-right VF vs horizontal axis | 1.58 | 0.12 | 0.17 | 0.0 |
| HVA vs VMA | 8.13 | <0.001 | <0.001 | 0.71 |
| Left-right VF vs upper-lower VF | -10.35 | <0.001 | <0.001 | -0.93 |
| Left-right VFI vs  non-meridian left-right VFI | -1.58 | 0.12 | 0.17 | -0.06 |
| Upper-lower VFI vs non-meridians upper-lower VFI | -0.79 | 0.43 | 0.43 | -0.02 |

*Table S3*. Formal comparisons of the differences. The comparisons were made by calculating the t-tests of the differences within the indices. FDR correction was applied to account for the multiple comparisons.

1. Accuracy, mean distance, and reaction time

Pearson’s correlation coefficients were calculated in order to test whether there is a link between the accuracy of the performance in the task and the average reaction time, as well as between the reaction time and the mean distance of all locations from the center throughout the last 5 trials. Both results did not yield statistical significance (r(260) = -0.12, p = 0.052 and p(26) = -0.1, p = 0.11 for the average reaction time and mean distance and accuracy and average reaction time respectively). For details, see Figure S3.


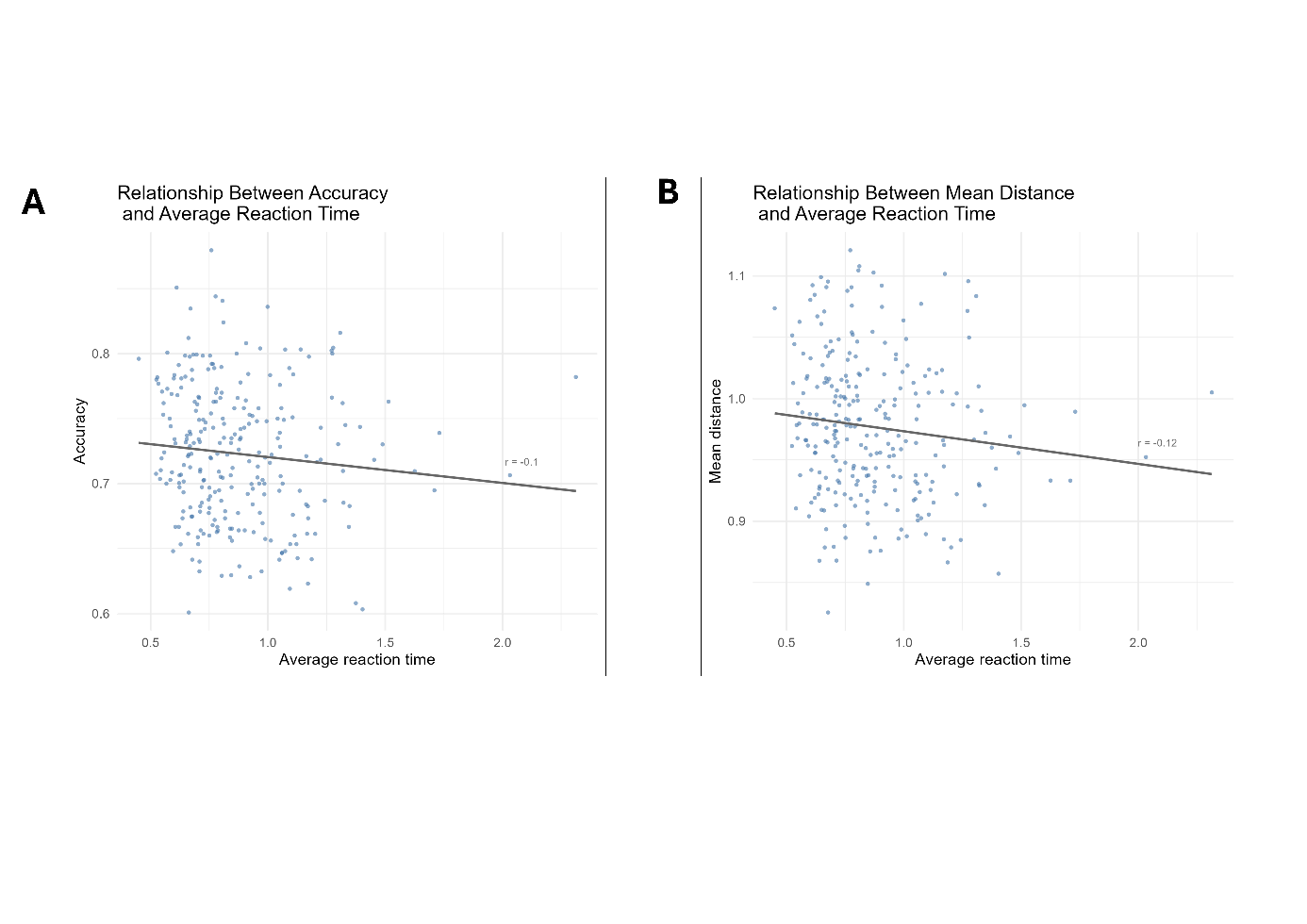


*Figure S3. A: scatter plot of accuracy in the task performance and the average reaction time of each participant. B: Scatter plot of the mean distance and average reaction time of each participant.*

1. Brain-behavior analyses including height as covariate

In line with the reasoning described in SI3, we cosidered if height should be added as a covariate into the volumetric models. To assess that, we ran a Pearson’s corelation coeffitient matrix to test whether any of used covariates share substantial variance. The results show moderate correlation of height with both sex and total intracranial volume (for details see Table S3). Taken together with the null correlations of height and behavioral indices, height was excluded as a covariate in the main brain analysis.

As a post-hoc robustness check, we re-estimated the main volumetric model for bilateral V1 and the VMA index including participant height as an additional covariate. The results are presented in Table S4. The overall pattern of results remained consistent with the primary analyses.

|  | Sex | Age | Total Intracranial Volume | Height |
| --- | --- | --- | --- | --- |
| Sex | 1 | -0.0765 | 0.5883 | 0.5573 |
| Age | -0.0765 | 1 | 0.0135 | 0.0310 |
| Total Intracranial Volume | 0.5883 | 0.0135 | 1 | 0.5032 |
| Height | 0.5573 | 0.0310 | 0.5032 | 1 |

*Table S3.* Pearson’s correlation coefficient matrix of covariates included in brain-behavior models.

|  | **Bilateral V1 multiparametrical maps** | | | | | | | |
| --- | --- | --- | --- | --- | --- | --- | --- | --- |
|  | **MT** | | **PD** | | **R1** | | **R2*** | |
| **Index** | χ2(1) | p | χ2(1) | p | χ2(1) | p | χ2(1) | p |
| Vertical Meridian Anisotropy (height included as a covariate) | 0.83 | 0.36 | 4.24 | 0.052 | 6.12 | 0.027 | 6.67 | 0.027 |

*Table S4.* Results of the Likelihood Ratio tests comparing more complex models (df = 7) explaining the variance of the ROI voxels against simpler ones (df = 6). The p-values were corrected for comparisons of different maps within the index using the FDR

1. Regions of interest

To further account for the retinotopic organization of V1 and to examine the spatial specificity of the observed effects, we repeated the primary likelihood ratio model comparisons within anatomically defined subdivisions of BA17. Specifically, BA17 was subdivided into left and right hemispheres to approximate contralateral visual hemifield representations, and into dorsal and ventral portions to approximate lower and upper visual field representations, respectively. In addition, quadrant-level subdivisions (left dorsal, left ventral, right dorsal, right ventral) were examined to further explore spatial specificity.

All analyses followed the same nested model comparison procedure described in the Methods section. For completeness, control analyses in BA18 (V2) and in a combined BA17+18 mask are also included in Table S4.

Across these subdivisions, significant effects were limited to the association between vertical meridian anisotropy and R2* within BA17. The observed association showed spatial specificity, being primarily localized to the ventral portion of left BA17. Effects in other subdivisions did not reach statistical significance

|  | **Left V1 multiparametrical maps** | | | | | | | | | | | | | |
| --- | --- | --- | --- | --- | --- | --- | --- | --- | --- | --- | --- | --- | --- | --- |
|  | **MT** | | **PD** | | | | **R1** | | | | **R2*** | | | |
| **Index** | χ2(1) | p | χ2(1) | | p | | χ2(1) | | p | | χ2(1) | | p | |
| Horizontal-Vertical Anisotropy | 0.94 | 0.953 | 0.83 | | 0.953 | | 0.38 | | 0.953 | | 0.03 | | 0.953 | |
| Vertical Meridian Asymmetry | 2.36 | 0.125 | **4.82** | | **0.038** | | **5.76** | | **0.033** | | **7.13** | | **0.03** | |
| Upper-Lower Visual Field Index | 3.63 | 0.227 | 1.78 | | 0.244 | | 1.16 | | 0.282 | | 2.3 | | 0.243 | |
| Left-Right Visual Field Index | 1.43 | 0.23 | 3.38 | | 0.13 | | 4.18 | | 0.13 | | 2.36 | | 0.17 | |
| Average Distance | 0.04 | 0.989 | 0.23 | | 0.989 | | 0.3 | | 0.989 | | <0.001 | | 0.989 | |
|  | **Right V1 multiparametrical maps** | | | | | | | | | | | | | |
|  | **MT** | | **PD** | | | | **R1** | | | | **R2*** | | | |
| **Index** | χ2(1) | p | χ2(1) | | p | | χ2(1) | | p | | χ2(1) | | p | |
| Horizontal-Vertical Anisotropy | 0.01 | 0.952 | 0.93 | | 0.952 | | 0.004 | | 0.952 | | 0.63 | | 0.952 | |
| Vertical Meridian Asymmetry | 0.06 | 0.814 | 2.68 | | 0.137 | | 4.82 | | 0.113 | | 2.67 | | 0.137 | |
| Upper-Lower Visual Field Index | 0.03 | 0.87 | 1.34 | | 0.493 | | 2.2 | | 0.493 | | 0.05 | | 0.870 | |
| Left-Right Visual Field Index | 0.27 | 0.89 | 0.18 | | 0.89 | | 1.63 | | 0.81 | | 0.02 | | 0.9 | |
| Average Distance | 1.63 | 0.404 | 0.36 | | 0.404 | | 0.06 | | 0.404 | | 2.4 | | 0.404 | |
|  | **Dorsal V1 multiparametrical maps** | | | | | | | | | | | | | |
|  | **MT** | | **PD** | | | | **R1** | | | | **R2*** | | | |
| **Index** | χ2(1) | p | χ2(1) | | p | | χ2(1) | | p | | χ2(1) | | p | |
| Horizontal-Vertical Anisotropy | < 0.001 | 0.994 | 0.94 | | 0.899 | | 0.14 | | 0.949 | | 0.57 | | 0.899 | |
| Vertical Meridian Asymmetry | 0.015 | 0.904 | 0.51 | | 0.633 | | 2.79 | | 0.189 | | 4.11 | | 0.170 | |
| Upper-Lower Visual Field Index | 1.82 | 0.355 | 2.18 | | 0.355 | | 0.77 | | 0.46 | | 0.55 | | 0.46 | |
| Left-Right Visual Field Index | 0.009 | 0.922 | 1.52 | | 0.433 | | 1.65 | | 0.433 | | 0.10 | | 0.922 | |
| Average Distance | 0.89 | 0.677 | 1.02 | | 0.677 | | 0.36 | | 0.677 | | 0.677 | | 0.677 | |
|  | **Ventral V1 multiparametrical maps** | | | | | | | | | | | | | |
|  | **MT** | | **PD** | | | | **R1** | | | | **R2*** | | | |
| **Index** | χ2(1) | p | χ2(1) | | p | | χ2(1) | | p | | χ2(1) | | p | |
| Horizontal-Vertical Anisotropy | 1.19 | 0.831 | 0.16 | | 0.831 | | 0.41 | | 0.831 | | 0.05 | | 0.831 | |
| Vertical Meridian Asymmetry | 2.12 | 0.145 | **5.24** | | **0.029** | | **6.15** | | **0.026** | | **6.34** | | **0.026** | |
| Upper-Lower Visual Field Index | 1.53 | 0.372 | 1.17 | | 0.372 | | 2.00 | | 0.372 | | 0.62 | | 0.431 | |
| Left-Right Visual Field Index | 0.52 | 0.472 | 1.16 | | 0.392 | | 3.16 | | 0.301 | | 1.10 | | 0.392 | |
| Average Distance | 0.65 | 0.836 | 0.24 | | 0.836 | | 0.003 | | 0.953 | | 0.73 | | 0.836 | |
|  | **Right Dorsal V1 multiparametrical maps** | | | | | | | | | | | | | |
|  | **MT** | | **PD** | | | | **R1** | | | | **R2*** | | | |
| **Index** | χ2(1) | p | χ2(1) | | p | | χ2(1) | | p | | χ2(1) | | p | |
| Horizontal-Vertical Anisotropy | 0.13 | 0.723 | 1.43 | | 0.464 | | 0.57 | | 0.599 | | 1.45 | | 0.464 | |
| Vertical Meridian Asymmetry | 0.41 | 0.656 | 0.20 | | 0.656 | | 2.65 | | 0.222 | | 2.54 | | 0.222 | |
| Upper-Lower Visual Field Index | 0.60 | 0.585 | 1.15 | | 0.585 | | 0.80 | | 0.585 | | 0.009 | | 0.924 | |
| Left-Right Visual Field Index | 0.50 | 0.638 | 1.44 | | 0.460 | | 2.32 | | 0.460 | | 0.007 | | 0.933 | |
| Average Distance | 1.93 | 0.568 | 0.36 | | 0.568 | | 0.33 | | 0.568 | | 0.76 | | 0.568 | |
|  | **Left Dorsal V1 multiparametrical maps** | | | | | | | | | | | | | |
|  | **MT** | | **PD** | | | | **R1** | | | | **R2*** | | | |
| **Index** | χ2(1) | p | χ2(1) | | p | | χ2(1) | | p | | χ2(1) | | p | |
| Horizontal-Vertical Anisotropy | 0.15 | 0.862 | 0.26 | | 0.862 | | 0.03 | | 0.862 | | 0.03 | | 0.862 | |
| Vertical Meridian Asymmetry | 0.38 | 0.539 | 1.34 | | 0.330 | | 2.36 | | 0.249 | | 4.36 | | 0.148 | |
| Upper-Lower Visual Field Index | 3.20 | 0.131 | 3.85 | | 0.131 | | 0.51 | | 0.476 | | 2.73 | | 0.131 | |
| Left-Right Visual Field Index | 0.79 | 0.497 | 1.42 | | 0.497 | | 0.46 | | 0.497 | | 0.60 | | 0.497 | |
| Average Distance | 0.007 | 0.934 | 1.96 | | 0.647 | | 0.29 | | 0.890 | | 0.18 | | 0.890 | |
|  | **Right Ventral V1 multiparametrical maps** | | | | | | | | | | | | | |
|  | **MT** | | **PD** | | | | **R1** | | | | **R2*** | | | |
| **Index** | χ2(1) | p | χ2(1) | | p | | χ2(1) | | p | | χ2(1) | | p | |
| Horizontal-Vertical Anisotropy | 0.02 | 0.922 | 0.48 | | 0.922 | | 0.13 | | 0.922 | | 0.01 | | 0.922 | |
| Vertical Meridian Anisotropy | 0.01 | 0.904 | 3.84 | | 0.100 | | 4.60 | | 0.100 | | 2.12 | | 0.194 | |
| Upper-Lower Visual Field Index | 0.03 | 0.865 | 0.99 | | 0.637 | | 2.33 | | 0.507 | | 0.15 | | 0.865 | |
| Left-Right Visual Field Index | 0.08 | 0.882 | 0.02 | | 0.882 | | 0.61 | | 0.882 | | 0.07 | | 0.882 | |
| Average Distance | 0.85 | 0.411 | 1.42 | | 0.411 | | 0.68 | | 0.411 | | 2.99 | | 0.335 | |
|  | **Left Ventral V1 multiparametrical maps** | | | | | | | | | | | | | |
|  | **MT** | | **PD** | | | | **R1** | | | | **R2*** | | | |
| **Index** | χ2(1) | p | χ2(1) | | p | | χ2(1) | | p | | χ2(1) | | p | |
| Horizontal-Vertical Anisotropy | 1.46 | 0.909 | 0.01 | | 0.911 | | 0.47 | | 0.911 | | 0.08 | | 0.911 | |
| Vertical Meridian Asymmetry | 3.11 | 0.078 | **4.85** | | **0.037** | | **5.64** | | **0.035** | | **6.86** | | **0.035** | |
| Upper-Lower Visual Field Index | 3.07 | 0.320 | 0.92 | | 0.336 | | 1.09 | | 0.336 | | 1.92 | | 0.332 | |
| Left-Right Visual Field Index | 1.38 | 0.241 | 3.20 | | 0.148 | | 4.95 | | 0.104 | | 2.59 | | 0.144 | |
| Average Distance | 0.07 | 0.912 | 0.01 | | 0.912 | | 0.22 | | 0.912 | | 0.01 | | 0.912 | |
|  | **V2 multiparametrical maps** | | | | | | | | | | | | | |
|  | **MT** | | **PD** | | | | **R1** | | | | **R2*** | | | |
| **Index** | χ2(1) | p | χ2(1) | | p | | χ2(1) | | p | | χ2(1) | | p | |
| Horizontal-Vertical Anisotropy | 0.13 | 0.885 | 0.02 | | 0.885 | | 0.03 | | 0.885 | | 0.62 | | 0.885 | |
| Vertical Meridian Asymmetry | 1.72 | 0.189 | 1.99 | | 0.189 | | 4.4 | | 0.071 | | 5.63 | | 0.07 | |
| Upper-Lower Visual Field Index | 3.73 | 0.214 | 1.86 | | 0.231 | | 1.9 | | 0.231 | | 0.7 | | 0.403 | |
| Left-Right Visual Field Index | 0.05 | 0.85 | 1.08 | | 0.6 | | 2.84 | | 0.37 | | 0.04 | | 0.85 | |
| Average Distance | 0.48 | 0.604 | 0.27 | | 0.604 | | 0.44 | | 0.604 | | 0.57 | | 0.604 | |
|  | **V1+V2 multiparametrical maps** | | | | | | | | | | | | | |
|  | **MT** | | | **PD** | | | | **R1** | | | | **R2*** | | |
| **Index** | χ2(1) | p | | χ2(1) | | p | | χ2(1) | | p | | χ2(1) | | p |
| Horizontal-Vertical Anisotropy | 0.29 | 0.795 | | 0.28 | | 0.795 | | 0.002 | | 0.966 | | 0.93 | | 0.795 |
| Vertical Meridian Asymmetry | 0.95 | 0.406 | | 0.69 | | 0.406 | | 3.09 | | 0.157 | | 5.3 | | 0.085 |
| Upper-Lower Visual Field Index | 3.57 | 0.236 | | 1.29 | | 0.416 | | 1.02 | | 0.416 | | 0.28 | | 0.594 |
| Left-Right Visual Field Index | 0.006 | 0.93 | | 0.59 | | 0.89 | | 2.46 | | 0.47 | | 0.02 | | 0.93 |
| Average Distance | 0.55 | 0.587 | | 0.3 | | 0.587 | | 0.33 | | 0.587 | | 0.76 | | 0.587 |

*Table S5.* Results of the Likelihood Ratio tests comparing more complex models (df = 7) explaining the variance of the ROI voxels against simpler ones (df = 6). The p-values were corrected for comparisons of different maps within each index using the FDR

1. Volumetric asymmetry indices

For completeness, we conducted additional analyses to examine whether visual hemifield asymmetries in behavior were reflected in corresponding structural asymmetries within V1. To this end, structural asymmetry indices were computed analogously to the behavioral indices, using mean MPM values extracted per participant from anatomically defined V1 subdivisions. Specifically, we denote:

V_dorsal_, V_ventral_, V_left_, V_right_

corresponding to the average MPM values within dorsal BA17, ventral BA17, left, and right BA17, respectively. Structural asymmetry indices were computed as follows:

$$Upper-Lower Structural Asymmetry = \frac{V_{dorsal}-V_{ventral}}{mean(V_{dorsal}, V_{ventral})} \times100$$

$$Left-Right Structural Asymmetry= \frac{V_{right}- V_{left}}{mean(V_{right}, V_{left})}\times100$$

The order of subtraction was chosen to mirror the behavioral indices and account for V1 retinotopy: ventral V1 corresponds to the upper visual hemifield, dorsal V1 to the lower visual hemifield, and each hemisphere represents the contralateral visual field. Descriptive statistics of the structural asymmetry indices are reported in Table S6.

Structural asymmetry indices were computed separately for each MPM contrast. Nested model comparisons were then performed following the same procedure as in the primary volumetric analyses, with the structural asymmetry index used as the dependent variable. The null model included total intracranial volume, age, and sex as nuisance covariates, along with a random intercept for participant. The alternative model additionally included the corresponding behavioral asymmetry index as a predictor. Results were corrected for multiple comparisons within each index using the FDR procedure.

No significant associations were observed between behavioral asymmetry indices and structural asymmetry measures (see Table S7).

|  | **V1 - volumetric asymmetry descriptive statistics** | | | | | | | | | | | | | | | |
| --- | --- | --- | --- | --- | --- | --- | --- | --- | --- | --- | --- | --- | --- | --- | --- | --- |
|  | **MT** | | | | **PD** | | | | **R1** | | | | **R2*** | | | |
| **Index** | Mean | SD | Min | Max | Mean | SD | Min | Max | Mean | SD | Min | Max | Mean | SD | Min | Max |
| Upper-Lower Visual Field Index | 14.35 | 9.37 | -1.99 | 47.08 | 4.30 | 3.28 | -3.51 | 16.91 | -4.34 | 4.71 | -21.87 | 3.94 | -41.43 | 11.62 | -84.83 | -14.13 |
| Left-Right Visual Field Index | -3.81 | 12.51 | -27.03 | 32.83 | -200.38 | 1.18 | -202.73 | -196.99 | -2.99 | 2.89 | -13.88 | 6.04 | 2.78 | 15.96 | -46.94 | 38.24 |

*Table S6*. Descriptive statistics of the volumetric asymmetry indices.

|  | **V1 - volumetric asymmetry analysis** | | | | | | | |
| --- | --- | --- | --- | --- | --- | --- | --- | --- |
|  | **MT** | | **PD** | | **R1** | | **R2*** | |
| **Index** | χ2(1) | p | χ2(1) | p | χ2(1) | p | χ2(1) | p |
| Upper-Lower Visual Field Index | 0.34 | 0.566 | 0.46 | 0.566 | 1.28 | 0.566 | 0.33 | 0.566 |
| Left-Right Visual Field Index | 1.95 | 0.190 | 1.83 | 0.190 | 1.75 | 0.190 | 3.09 | 0.190 |

*Table S7.* Results of the Likelihood Ratio tests comparing more complex models (df = 7) explaining the variance of the ROI voxels against simpler ones (df = 6). The p values were corrected for comparisons of different maps within each index using the FDR.

1. References

Carrasco M, Myers C, Roberts M. 2023. Visual field asymmetries vary between adolescents and adults. [accessed 2024 June 3]. http://biorxiv.org/lookup/doi/10.1101/2023.03.04.531124. <https://doi.org/10.1101/2023.03.04.531124>

Carrasco M, Myers C, Roberts M. 2025. Visual field asymmetries develop throughout adolescence. iScience. 28(11):113782. <https://doi.org/10.1016/j.isci.2025.113782>

Carrasco M, Roberts M, Myers C, Shukla L. 2022. Visual field asymmetries vary between children and adults. Curr Biol. 32(11):R509–R510. https://doi.org/10.1016/j.cub.2022.04.052

Rossion B, Pourtois G. 2004. Revisiting Snodgrass and Vanderwart’s Object Pictorial Set: The Role of Surface Detail in Basic-Level Object Recognition. Perception. 33(2):217–236. https://doi.org/10.1068/p5117
